# Editorial: Nutritional challenges and therapies in youth with chronic inflammatory diseases

**DOI:** 10.3389/fnut.2025.1740365

**Published:** 2026-01-13

**Authors:** Patrícia Zamberlan, Jane Oba, Artur Figueiredo Delgado

**Affiliations:** Faculty of Medicine, University of São Paulo, São Paulo, Brazil

**Keywords:** nutrition, chronic disease, inflammation mediators, body composition, molecular targeted therapy

## Introduction

Nutrition has moved far beyond its traditional role of providing energy and essential nutrients. It is now recognized as a core component of precision medicine, capable of modulating inflammation, metabolism, and treatment response in chronic diseases. The studies gathered in this issue of *Frontiers in Nutrition* illustrate how advances in biomarkers, body composition assessment, and phytochemical research are redefining the boundaries of clinical nutrition.

### Inflammation and metabolic regulation

The inflammatory–nutritional axis is increasingly recognized as a key determinant in chronic disease (1), and two studies in this issue highlight this relationship. The first shows that a high Systemic Immune-Inflammation Index (SII) predicts central obesity in children, even before fat accumulation becomes evident (Zhang et al.). This finding reframes obesity as an immune-metabolic condition, emphasizing the value of early nutritional interventions to prevent inflammation-driven adiposity.

In adults, the Advanced Lung Cancer Inflammation Index (ALI) demonstrates a nonlinear “J-shaped” association with mortality in patients with chronic airway disease (Luo et al.). Both undernutrition and obesity-related inflammation were linked to increased mortality. Together, these findings reinforce that the goal of nutritional care is not to normalize body weight alone, but to restore the inflammatory–nutritional balance that supports survival and metabolic health.

### Body composition and therapeutic outcomes

Beyond inflammation, body composition has emerged as a determinant of treatment efficacy and prognosis (2). In children with Crohn's disease, the presence of sarcopenia—measured by CT-derived skeletal muscle index—was associated with twice the risk of losing response to biologic therapy (Guo et al.).

This underscores that muscle mass is not merely structural, but a metabolic and immunologic organ influencing drug metabolism and inflammatory control. Nutritional support through exclusive or partial enteral nutrition improved muscle status, although residual sarcopenia persisted in some patients, highlighting the need for integrated therapy combining nutrition, pharmacology, and exercise.

### Phytochemicals and molecular nutrition

Several studies explore nutritional modulation at the molecular level. Supplementation with an aqueous extract of *Quercus brantii* (Iranian oak) improved lipid profiles in beta-thalassemia patients without adverse hepatic effects (Satehi et al.). Its polyphenols counter oxidative and lipid damage induced by iron overload—demonstrating the potential of nutritional adjuvants alongside standard pharmacologic treatments.

In an experimental study, the soy isoflavone Biochanin A promoted the formation of type H vessels and improved bone regeneration in juvenile mice after osteonecrosis (Huang et al.). Acting through Platelet-Derived Growth Factor-BB (PDGF-BB) signaling, it redirected osteoclast precursors toward angiogenesis and repair. This represents an innovative concept of nutritional tissue regeneration, where bioactive compounds influence cellular differentiation and healing.

### Operational and global perspectives

Scientific advances are only meaningful if effectively implemented in clinical care. The study on interruptions in nutritional therapy among critically ill children with chronic diseases revealed that nearly half were undernourished, and feeding interruptions were frequent, often due to logistical issues rather than intolerance (Zamberlan et al.).

Such findings emphasize that the success of nutrition therapy depends on continuity and operational efficiency. Protocols such as volume-based feeding and adequate use of parenteral nutrition are essential to avoid iatrogenic deficits and achieve energy goals (3).

On a global scale, the International Initiative for Pediatrics and Nutrition (IIPAN) provides consensus recommendations for the nutritional management of children with cancer in resource-limited settings (Viani et al.). The guidelines adapt anthropometric tools and intervention strategies to diverse clinical contexts, proving that precision nutrition can also be equitable and feasible in low- and middle-income countries. These recommendations, aligned with WHO and International Society of Pediatric Oncology (ISPO) initiatives, mark a crucial step toward closing global gaps in pediatric nutritional care (4).

### Emerging frontiers: obesity, asthma, and the gut–lung axis

A bibliometric analysis of obesity-related asthma research (2004–2023) reveals a growing focus on microbiota, systemic inflammation, and the gut–lung axis (Lang et al.). This trend signals the next frontier in nutritional science—integrating dietary modulation of the microbiome to influence immune and respiratory outcomes. Nutrition thus becomes a tool to modify not only metabolism but also host–microbe interactions underlying chronic inflammation.

## Conclusion

Collectively, the studies in this issue advance a unified view of nutrition as a precision-based medical intervention. Whether through inflammatory biomarkers, body composition targets, or bioactive compounds, nutrition demonstrates its potential to influence disease mechanisms at multiple levels.

Four key directions emerge for clinical and research practice:

- Incorporate inflammatory and muscle biomarkers into risk assessment and monitoring;
- Ensure continuity and safety of nutritional therapy, especially in critical care;
- Expand research on nutraceuticals and phytochemicals with molecular and regenerative effects;
- Implement context-specific guidelines that bridge scientific precision and health equity;

By uniting inflammation control, metabolic modulation, and molecular repair, nutrition stands as a central pillar of precision medicine, capable of transforming prevention, treatment, and recovery across chronic diseases (5, 6).
